# Impact of various solutions on the oral health status of critically ill patients

**DOI:** 10.25122/jml-2023-0495

**Published:** 2024-03

**Authors:** Shaimaa Ahmed Awad Ali, Nourah Alsadaan, Mariam Ameer, Mohamed Sayed-Ahmed, Fahad Alanazi

**Affiliations:** 1 Medical -Surgical Department, College of Nursing, Jouf University, Sakaka, Al-Jouf, Saudi Arabia; 2 College of Nursing, Jouf University, Sakaka, Al-Jouf, Saudi Arabia; 3 Department of Physical Therapy and Health Rehabilitation, College of Applied Medical Sciences, Jouf University, Sakaka, Al-Jouf, Saudi Arabia; 4 Department of Biomechanics, Faculty of Physical Therapy, Cairo University, Giza, Egypt; 5 Department of Clinical Pharmacy, College of Pharmacy, Jazan University, Jazan, Saudi Arabia; 6 Department of Internal Medicine and Infectious Disease, Faculty of Veterinary Medicine, Mansoura University, Mansoura, Egypt

**Keywords:** oral health care, chlorhexidine gluconate, hexetidine, critically ill patients, oropharyngeal colonization, tracheal colonization, pulmonary infections, normal saline

## Abstract

Oral care is a crucial challenge of nursing care in orally intubated patients. Oropharyngeal colonization with microorganisms is probably the first step in the pathogenesis of most bacterial pulmonary infections. This study aimed to investigate the effect of different oral care solutions on the oral health status of critically ill patients. We conducted a quasi-experimental study involving a convenience sample of 60 adult orally intubated patients, distributed equally into three groups: 20 patients received 0.12% chlorhexidine gluconate (CHX) solution as an oral rinse; 20 patients received 0.1% hexetidine (HEX) solution as an oral rinse; and a control group of 20 patients received routine hospital oral care with 0.9% normal saline (NS) solution. Oropharyngeal and tracheal cultures were obtained from patients within 24–48 h of admission, before the administration of topical oral antimicrobial solutions and then repeated on day 4 and day 7 after the oral solutions. The study revealed that CHX has a more powerful effect than HEX and NS in improving the oral mucosa and decreasing colonization of both the oropharynx and trachea. On day 7, the improvements were statistically significant in the CHX group and the HEX group (*P* = 0.02 and *P* = 0.03, respectively), but not in the NS group. This research confirms the effect of CHX and HEX in lowering the risk of tracheal and oropharyngeal colonization, and recommends the use of a CHX solution as oral mouth care in critically ill patients.

## INTRODUCTION

During a hospital stay, oral health plays an important role in the care plan of the patient. Nevertheless, research suggests that the patients’ dental health frequently deteriorates throughout their hospital stay, especially in the case of patients who need mechanical ventilation [1]. Given that plaque can build in as little as 48 h, the oral health of these patients can rapidly deteriorate owing to the extended periods of time when their mouth is open and to their dependency on clinical professionals for oral care [2]. In addition to promoting oral health, oral care eases patient discomfort, and reduces mucosal irritation and tooth plaque.

Hospital-acquired infections is a major issue in critically ill patients. Studies have shown that combined with other preventive measures, dental hygiene can help lower the risk of ventilator-associated pneumonia (VAP) in intubated patients [3]. The main objective of oral care is to attenuate bacterial plaque formation and buildup of oropharyngeal debris, which causes conditions such as stomatitis and periodontitis. The presence of plaque for more than 3 days increases the number of Gram-negative microorganisms in the oral cavity. These microorganisms can cause infection not only in the oral cavity but other organs as well, leading to pneumonia, meningitis, mediastinal abscess, osteomyelitis, cardiovascular disease, endocarditis, and bacteremia [4–6]. However, compared to other areas of care, delivering dental care for patients in the intensive care unit (ICU) is particularly challenging and receives less attention from medical professionals [7].

Researchers have studied three aspects of oral hygiene: the use of assessment tools, the administration of cleansing agents or chemicals, and the frequency of oral hygiene procedures. Several nursing studies acknowledge the importance of assessment tools; however, nursing practices continue to be guided by ritual, tradition, and the individual preferences of nurses [8]. In the ICU, ventilator-associated pneumonia (VAP) is the most frequent nosocomial infection [9], and the aspiration of microorganisms from the oral cavity as a result of inadequate oral hygiene practices is one of the main factors contributing to VAP [2]. The most frequently isolated pathogens in individuals with VAP are *Pseudomonas aeruginosa, Escherichia coli, Klebsiella pneumoniae, Staphylococcus* species, and *Acinetobacter* species [10].

Mouthwashes are liquids or solutions that are used to rinse the oral cavity for various reasons, such as deodorizing, eliminating or destroying microorganisms, and providing medicinal benefits by reducing infection or preventing dental caries. Based on their pharmacological characteristics, these topical antibiotics can be categorized into two groups or generations. First-generation compounds, such as sanguinarine, are capable of killing bacteria upon contact, but their ability to modify the oral flora following expectoration is restricted. Second-generation agents, such as chlorhexidine gluconate (CHX), have a longer-lasting impact on the oral flora in addition to their rapid antibacterial action [11].

The most common antiseptic agents used in mouthwashes are CHX and hexetidine (HEX). CHX is a compound with broad-spectrum antimicrobial activity, being efficient against Gram-positive and Gram-negative bacteria, as well as yeasts [12]. A study involving patients who had been intubated for more than 72 h found that 57% of them had bacteria that were resistant to many antibiotics, which would probably increase the expense of their care [13]. Several recently published clinical trials have shown that intra-oral disinfection with topical CHX and teeth brushing can reduce the rate of VAP in patients with mechanical ventilation and the prevalence of oropharyngeal colonization. However, these studies did not compare the effectiveness of CHX with other oral care solutions [14,15].

The aim of this study was to investigate the effect of different oral rinses on reducing oropharyngeal and tracheal colonization and enhancing the oral health status of critically ill, intubated patients.

## MATERIAL AND METHODS

We performed a quasi-experimental study at the emergency hospital’s ICUs at Mansoura University. A convenience sample of 60 adult, orally intubated patients admitted to the previously mentioned setting was studied. All included patients were aged between 21 and 60 years, and had endotracheal tubes that were either attached to a mechanical ventilator or oxygen source via a t-piece. The expected time in ICU was more than 6 days, intubation was performed within 24 h of ICU admission, and the interval between intubation and first microbial culture was less than 48 h. Patients who were receiving chemotherapy or radiotherapy to the head and neck, as well as patients with diabetes, severe liver or kidney disease, or autoimmune disease were excluded from the study.

### Data collection tools

After reviewing the related literature, we used two tools for data collection. The first one was the oral assessment tool, consisting of two parts, a patient’s profile sheet and an oral assessment sheet. The patient’s profile sheet included patient characteristics such as age and sex, whereas the oral assessment sheet included the assessment of six components (lips, tongue, saliva, mucous membrane, gingiva, and teeth). Each scale was scored from 1 to 3, yielding a maximum total score of 18 after the examination of the six subscales. Scores between 6 and 10 indicated minor modifications, and scores between 11 and 18 indicated major modifications. The examinations were carried out on day 1, 4, and 7 after enrollment.

The second tool consisted in an oral care sheet that was developed by the authors after reviewing the related literature [12]. This tool was used to compile data regarding the baseline oropharyngeal and tracheal cultures that were obtained from patients within 24–48 h of admission, before the administration of topical oral antimicrobial solutions using an oral foam applicator saturated with rinse solution and then repeated on day 4 and day 7. Oral care was performed using a soft pediatric toothbrush for brushing the teeth, gums, and tongue with antimicrobial solutions.

### Fieldwork

A pilot study was conducted on 10% of the sample to test the applicability of the tools. On admission, the patients were randomly allocated into three equal groups: 20 patients received 0.12% CHX solution as an oral rinse; 20 patients received 0.1% HEX solution as an oral rinse; and a control group of 20 patients received routine hospital oral care with 0.9% NS solution. In each group, oral care was performed every 8 h daily, for 6 days.

A baseline oral assessment was done for all patients on admission. The patients’ lips, tongue, saliva, mucous membrane, and teeth were assessed using the oral assessment tool, and the assessment was repeated on day 4 and day 7 of admission. A comparison of the differences between the three assessments of oral status within each group and among the three groups was done.

Oropharyngeal swabs and tracheal aspirates were obtained from patients in the morning, within 24–48 h after admission, before the application of the oral rinse solutions, and were repeated on day 4 and day 7. For the collection of oropharyngeal swabs, the tongue was gently depressed with a tongue depressor and the swab was introduced to the patient’s oropharynx, which was rubbed in a circular motion with the swab. Care was taken not to touch the sidelong walls of the oral cavity or the tongue. Tracheal aspirates were collected by inserting a sterile catheter into the endotracheal tube for a minimum of 30 cm without suction. After the positioning of the catheter, the aspirates were collected into a sterile container. A comparison of the differences between the culture of samples obtained on day 1, 4, and 7 within each group and among the three groups was done.

### Oral care technique

The patient’s head was placed to one side or placed in a semi-Fowler’s position. Deep suction was provided as needed. The oropharyngeal airway was removed, cleaned, and replaced after mouth care was performed. The teeth were brushed according to the recommendations of the American Dental Association. Four dental quadrants (upper right, lower right, upper left, and lower left) were identified, and a specific brushing pattern was applied to each quadrant. Every tooth in each quadrant had its lingual, buccal, and biting surfaces brushed five times. Using a soft pediatric toothbrush and a flexible hand to fit around the endotracheal tube, the teeth were cleaned for 1–2 min.

In total, 15 ml of CHX and HEX were used in the corresponding groups, using an oral foam applicator saturated with rinse solution (Sage Products) every 8 h, daily for 6 days. The solutions were applied for 1 min. The toothbrush was held at a 45° angle, and small circular or horizontal strokes were made with light pressure. To remove debris, the endotracheal tube was gently scrubbed with a toothbrush and gauze as part of dental care. The replacement was done side to side. Excess fluids and secretions were removed from the oral cavity with a suction device, and the gums and tongue were lightly cleaned to promote tissue perfusion and remove any debris. A lip lubricant was applied to moisten the lips. A comparison between the three groups was made to determine which solution was the most effective in reducing the number of pathogens.

### Statistical analysis

Microsoft Excel (Microsoft) and SPSS 20 (IBM Corp) were used to perform the statistical analysis of the data. Descriptive analysis was performed to examine frequency and proportion, and calculate means ± s.d. Statistically significant differences among the three groups were examined using Student’s *t*-test for quantitative data, and the chi-squared test for qualitative data. A significance threshold of *P* < 0.05 with a 95% confidence interval was chosen for the investigation.

## RESULTS

In terms of patient characteristics, the age range for 60%, 55%, and 50% of the patients in the CHX, HEX, and NS groups was between 21 and 40 years, whereas the age range for 40%, 45%, and 50% of the patients in the three groups was between 41 and 60 years. In terms of gender, 55% of the HEX group, and 65% of the NS and CHX groups were men. Concerning the level of education, more than half of all patients had basic education, not reaching secondary school level. There were no significant differences in general characteristics among the three groups (Table 1).

**Table 1:** Patient characteristics

Characteristics	CHX (*n* = 20)		HEX (*n* = 20)		NS (*n* = 20)		CHX vs. HEX		CHX vs. NS		HEX vs. NS	
	*n*	%	*n*	%	*n*	%	Χ^2^	*P* value	Χ^2^	P value	Χ^2^	*P* value
**Age (years)**												
21–40	12	60	11	55	10	50	0.11	0.74	0.1	0.75	0.01	0.9
41–60	8	40	9	45	10	50						
**Sex**												
Male	13	65	11	55	13	65	0.41	0.52	0.001	1	0.41	0.75
Female	7	35	9	45	7	35						
**Education**												
>2ry	9	45	8	40	8	40	0	0.75	0.1	0.75	0.001	1
<2ry	11	55	12	60	12	60						
**Residence**												
Urban	9	45	7	35	8	40	0.42	0.52	0.1	0.75	0.11	0.74
Rural	11	55	13	65	12	60						

On day 1 and day 4, there were no significant differences among the three groups. However, on day 7, 75% of patients in the CHX group showed mild alteration of the oral cavity compared with 70% in the HEX group and 30% in the NS group. The difference between the CHX and the NS group (*P* = 0.01) and between the HEX and the NS group (*P* = 0.02) was statistically significant. On day 7, there was a significant difference compared to baseline both within the CHX (*P* = 0.02) and the HEX group (*P* = 0.03), but not in the NS group (Table 2).

**Table 2:** Relationship between oral assessment and oral rinses on day 1, 4, and 7

Oral assessment		CHX (*n = 20*)		HEX (*n = 20*)		NS (*n = 20*)		CHX vs. HEX		CHX vs. NS		HEX vs. NS	
		*n*	%	*n*	%	*n*	%	Χ^2^	*P* value	Χ^2^	*P* value	Χ^2^	*P* value
Day 1 (before rinsing)	Mild alteration	8	40	7	35	9	45	0.1	0.75	0.1	0.75	0.63	0.52
	Severe alteration	12	60	13	65	11	55						
Day 4	Mild alteration	9	45	9	45	8	40	0.001*	1	0.1	0.75	0.1	0.75
	Severe alteration	11	55	11	55	12	60						
Day 7	Mild alteration	15	75	14	70	6	30	0.12	0.24	6.4	0.01*	4.9	0.02*
	Severe alteration	5	25	6	30	14	70						
Day 1 vs. day 4		*P* = 0.75		*P* = 0.75		*P* = 0.75							
Day 1 vs. day 7		*P* = 0.02^*^		*P* = 0.03^*^		P = 0.3							

* Statistically significant

On admission, *Streptococcus* and *Staphylococcus aureus* were the most frequently isolated microorganisms in the three groups. On day 4, there was an increase in the number of patients with *Klebsiella* and *Proteus*, which remained high until day 7 in the NS group. However, in the CHX group, the number of patients with *Streptococcus, Staphylococcus*, and *Klebsiella* infection has reduced from day 4 to day 7. A significant difference was found between the CHX and the NS group and between the HEX and the NS group on day 7, as *Klebsiella* was isolated in 45% of the NS group, 10% of the CHX group (*P* = 0.013), and 15% of the HEX group (*P* = 0.04) (Table 3).

**Table 3:** Frequency of oropharyngeal colonization with different bacterial species among the three groups on day 1, 4, and 7

Oropharyngeal bacterial species	Day	CHX (n = 20)		HEX (n = 20)		NS (n = 20)		CHX vs. HEX		CHX vs. NS		HEX vs. NS	
		*n*	%	*n*	%	*n*	%	Χ^2^	*P* value	Χ^2^	*P* value	Χ^2^	*P* value
**Gram–positive**													
*Streptococcus*	1	5	25	4	20	5	25	0.98	1.0	0.001	1	0.98	1.0
	4	4	20	4	20	–	–	0.001	1.0	0.98	0.1	0.98	0.1
	7	–	–	1	5	–	–	0.98	1.0	0.001	1.0	0.98	1.0
*Staphylococcus aureus*	1	4	20	4	20	4	20	0.001	1.0	0.001	1.0	0.001	1.0
	4	3	15	4	20	4	20	0.98	1.0	0.98	1.0	0.001	1.0
	7	2	10	3	15	5	25	0.98	1.0	1.6	0.4	2.02	0.7
*Actinomyces*	1	1	5	1	5	1	5	0.001	1.0	0.001	1.0	0.001	1.0
	4	1	5	1	5	1	5	0.001	1.0	0.001	1.0	0.001	1.0
	7	1	5	1	5	1	5	0.001	1.0	0.001	1.0	0.001	1.0
**Gram–negative**													
*Klebsiella*	1	3	15	3	15	3	15	0.001	1.0	0.001	1.0	0.001	1.0
	4	2	10	3	15	8	40	0.98	1.0	4.8	0.03*	2.98	0.08
	7	2	10	3	15	9	45	0.98	1.0	6.01	0.013*	4.18	0.04*
*Proteus*	1	3	15	4	20	3	15	0.98	1.0	0.001	1.0	0.98	1.0
	4	3	15	3	15	4	20	0.001	1.0	0.98	1.0	0.98	1.0
	7	–	–	1	5	5	25	0.98	1.0	5.74	0.047*	1.76	0.18
*Escherichia coli*	1	2	10	2	10	2	10	0.001	1.0	0.001	1.0	0.001	1.0
	4	2	10	2	10	2	10	0.001	1.0	0.001	1.0	0.001	1.0
	7	1	5	1	5	2	10	0.001	1.0	0.98	1.0	0.98	1.0
*Pseudomonas*	1	–	–	–	–	–	–	0.001	1.0	0.001	1.0	0.001	1.0
	4	–	–	1	5	7	35	0.98	1.0	8.48	0.008*	3.41	0.04*
	7	–	–	1	5	7	35	0.98	1.0	8.48	0.008*	3.41	0.04*

* Statistically significant

In total, 60% of patients tested negative for bacterial infection in the CHX group (*P* = 0.001), and 35% tested negative in the HEX group (*P* = 0.008). Furthermore, the number of patients infected with oropharyngeal Gram-positive microorganisms was reduced by 70% in the CHX group (*P* = 0.02). As far as Gram-negative microorganisms are concerned, the number of patients infected with these pathogens was reduced by 50% (*P* = 0.1) in the CHX group and 27% in the HEX group; however, there was a 90% increase in the number of patients infected with Gram-negative microorganisms in the NS group (*P* = 0.001). A highly significant difference was found between the CHX and the NS group (*P* = 0.001) and between the HEX group and NS group (*P* = 0.001) regarding the isolation of oropharyngeal Gram-negative microorganisms (Table 4).

**Table 4:** Distribution of oropharyngeal cultures among the three groups before and after 7 days of using oral rinse solutions

Oropharyngeal cultures	CHX (*n* = 20)		HEX (*n* = 20)		NS (n = 20)		CHX vs. HEX		CHX vs. NS		HEX vs. NS	
	*n*	%	*n*	%	*n*	%	X^2^	*P* value	X^2^	*P* value	X^2^	*P* value
**Negative cultures**												
Before	–	–	–	–	–	–	–	–	–	–	–	–
After	12	60	7	35	1	5	2.5	0.11	11.4	0.001	3.91	0.044
Before vs. after	X^2^ = 17.14 *P* = 0.001^*^		X^2^ = 6.23 *P* = 0.008^*^		X^2^ = 0.98 *P* = 1.0							
**Gram-positive organisms**												
Before	10	50	9	45	10	50	0.1	0.75	0.001	1	0.1	0.75
After	3	15	5	25	6	30	0.15	0.69	0.31	0.45	0.1	0.72
Before vs. after	X^2^ = 4.1 *P* = 0.02^*^		X^2^ = 0.91 *P* = 0.18		X^2^ = 0.86 *P* = 0.2							
**Gram-negative organisms**												
Before	10	50	11	55	10	50	0.1	0.75	0.001	1	0.1	0.75
After	5	25	8	40	19	95	1.03	0.31	17.6	0.001*	11.4	0.001*
Before vs. after	X^2^ = 2.67 P = 0.1		X^2^ = 1.3 P = 0.34		X^2^ = 10.16 P = 0.001^*^							

* Statistically significant

On day 4, there was a reduction in the number of patients with bacterial infections. On day 7, the most frequently isolated microorganisms in the NS group were *Klebsiella* (70%), *Pseudomonas* (35%), *Proteus* (30%), and *Staphylococcus aureus* (30%). However, there was a reduction in the number of patients with bacterial infections in the study groups, and two microorganisms, *Streptococcus* and *Staphylococcus aureus*, disappeared completely. Also on day 7, significant differences were found between the CHX and the NS group, and between the HEX and the NS group regarding the isolation of *Klebsiella* and *Staphylococcus aureus*, whereas *Pseudomonas* was present in 35% of patients in the NS group and 0% of patients in the CHX group (*P* = 0.008) (Table 5).

**Table 5:** Frequency of tracheal colonization with different bacterial species among the three groups on day 1, 4, and 7

Oropharyngeal bacterial species	Day	CHX (*n* = 20)		HEX (*n* = 20)		NS (*n* = 20)		CHX vs. HEX		CHX vs. NS		HEX vs. NS	
		*n*	%	*n*	%	*n*	%	Χ^2^	*P* value	Χ^2^	*P* value	Χ^2^	*P* value
**Gram-positive**													
*Streptococcus*	1	3	15	2	10	2	10	0.98	1.0	0.98	1.0	0.001	1.0
	4	2	10	–	–	–	–	0.53	0.5	0.53	0.5	0.001	1.0
	7	–	–	–	–	–	–	0.001	1.0	0.001	1.0	0.001	1.0
*Staphylococcus aureus*	1	3	15	3	15	3	15	0.001	1.0	0.001	1.0	0.001	1.0
	4	3	15	2	10	4	20	0.98	1.0	0.98	1.0	0.32	0.7
	7	–	–	–	–	6	30	0.001	1.0	4.9	0.02*	4.9	0.02*
*Actinomyces*	1	1	5	1	5	1	5	0.001	1.0	0.001	1.0	0.001	1.0
	4	1	5	1	5	3	15	0.001	1.0	0.28	0.6	0.28	0.6
	7	1	5.3	1	5	3	15	0.001	1.0	0.28	0.6	0.28	0.6
**Gram-negative**													
*Klebsiella*	1	4	20	4	20	3	15	0.001	1.0	0.98	1.0	0.98	1.0
	4	2	10	5	25	9	45	0.69	0.4	6.11	0.013*	1.76	0.18
	7	1	5.3	5	25	14	70	0.98	0.1	18.1	0.001*	8.12	0.004*
*Enterobacter*	1	–	–	–	–	–	–	0.001	1.0	0.001	1.0	0.001	1.0
	4	–	–	–	–	1	5	0.001	1.0	0.98	1.0	0.98	1.0
	7	–	–	–	–	1	5	0.001	1.0	0.98	1.0	0.98	1.0
*Proteus*	1	3	15	4	20	4	20	0.98	1.0	0.98	1.0	0.001	1.0
	4	2	10	4	20	5	25	0.32	0.7	0.69	0.4	0.98	1.0
	7	1	5.3	1	5.3	6	30	0.001	1.0	2.77	0.09	2.77	0.09
*Escherichia coli*	1	4	20	4	20	4	20	0.001	1.0	0.001	1.0	0.001	1.0
	4	5	25	4	20	4	20	0.98	1.0	0.98	1.0	0.001	1.0
	7	3	15	3	15	4	20	0.001	1.0	0.98	1.0	0.98	1.0
*Pseudomonas*	1	–	–	–	–	–	–	0.001	1.0	0.001	1.0	0.001	1.0
	4	–	–	2	10	4	20	0.49	0.5	1.03	0.11	1.21	0.66
	7	–	–	2	10	7	35	0.49	0.5	8.3	0.008*	0.89	0.13
*Citrobacter*	1	2	10	2	10	2	10	0.001	1.0	0.001	1.0	0.001	1.0
	4	2	10	2	10	3	15	0.001	1.0	0.98	1.0	0.98	1.0
	7	2	10	2	10	3	15	0.001	1.0	0.98	1.0	0.98	1.0

* Statistically significant

In total, 25% of the patients tested negative for any bacterial species after CHX and HEX treatment (*P* = 0.047). The number of patients infected with endotracheal Gram-positive microorganisms was reduced 85.7% in the CHX group (*P* = 0.044). However, the number of patients infected with Gram-positive microorganisms was reduced 83.3% in the HEX group and was increased 16.6% in the NS group. Significant differences were found between the CHX and the NS group (*P* = 0.04) and between the HEX and the NS group (*P* = 0.04) regarding the isolation of Gram-positive microorganisms. Regarding Gram-negative microorganisms, there was a 46.15% reduction in the number of patients infected with Gram-negative microorganisms in the CHX group (*P* = 0.058) compared to a 7.14% reduction in the HEX group. However, there was a 35.7% increase in the number of patients infected with Gram-negative microorganisms in the NS group. A significant difference was found between the CHX and the NS group (*P* = 0.001) and between the HEX and the NS group (*P* = 0.044) regarding the isolation of endotracheal Gram-negative microorganisms (Table 6).

**Table 6:** Distribution of endotracheal cultures among the three groups before and after 7 days of using oral rinse solutions

Oropharyngeal cultures	CHX (*n* = 20)		HEX (*n* = 20)		NS (*n* = 20)		CHX vs. HEX		CHX vs. NS		HEX vs. NS	
	*n*	%	n	%	*n*	%	Χ^2^	*P* value	Χ^2^	*P* value	Χ^2^	*P* value
**Negative cultures**												
Before	–	–	–	–	–	–	–	–	–	–	–	–
After	5	25	5	25	1	5	0.001	1.0	1.67	0.2	1.67	0.2
Before vs. after	X^2^ = 2.68 *P* = 0.047^*^		X^2^ = 2.68 *P* = 0.047^*^		X^2^ = 0.001 *P* = 1.0							
**Gram-positive organisms**												
Before	7	35	6	30	6	30	0.1	0.74	0.1	0.74	0.001	1.0
After	1	5	1	5	7	35	0.001	1.0	3.91	0.04*	3.91	0.04*
Before vs. after	X^2^ = 3.1 *P* = 0.044^*^		X^2^ = 2.8 *P* = 0.09		X^2^ = 0.1 *P* = 0.74							
**Gram-negative organisms**												
Before	13	65	14	70	14	70	0.1	0.74	0.1	0.74	0.001	1.0
After	7	35	13	65	19	95	2.6	0.06	15.8	0.001*	6.72	0.044*
Before vs. after	X^2^ = 2.98 *P* = 0.058^*^		X^2^ = 0.1 *P* = 0.74		X^2^ = 2.8 *P* = 0.09							

* Statistically significant

There were microbiological similarities between microorganisms infecting the trachea and oropharyngeal flora: it can be noted that there was a concordance between microorganisms infecting the trachea and oropharyngeal flora. Before the use of solutions, 40% of both Chlorhexidine and Hexetidine groups and 25% of the N.S group had similar organisms infecting both the trachea and oropharynx. On day 7, there were 95% similarities between microorganisms infecting the trachea and oropharyngeal flora in the NS group, 20% in the HEX group, and 15% in the CHX group (Table 7).

**Table 7:** Microbiological similarities between microorganisms infecting the trachea and oropharyngeal flora

Similarities between oropharyngeal and tracheal flora	Day	CHX (*n* = 20)		HEX (*n* = 20)		NS (*n* = 20)	
		*n*	%	*n*	%	*n*	%
**Similar organism**	1	8	40	8	40	5	25
	4	7	35	7	35	14	70
	7	3	15	4	20	19	95
**Different organism**	1	13	65	13	65	15	75
	4	9	45	13	65	17	85
	7	4	20	9	45	17	85

**Figure 1:**
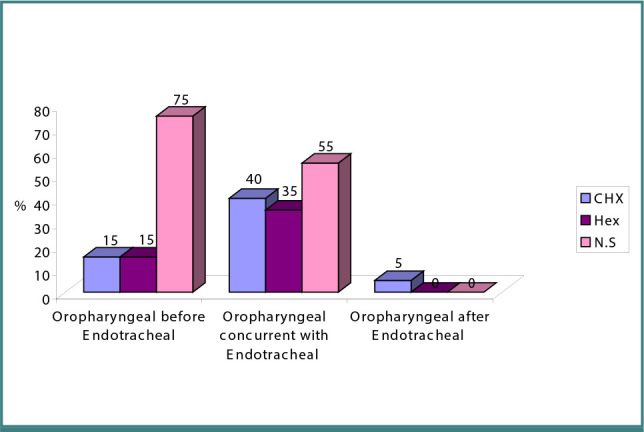
Frequency of oropharyngeal colonization and tracheal colonization among the three groups

The frequency of oropharyngeal and tracheal colonization among the three oral rinses groups is presented in Figure 1. It can be noted that oropharyngeal colonization preceded tracheal colonization in 15% of both the CHX and HEX groups and 75% of the NS group. It can also be seen that 40% of the CHX group, 35% of the HEX group, and 55% of the NS group had oropharyngeal colonization concurrent with tracheal colonization. Only one patient (5%) in the CHX group had oropharyngeal colonization after tracheal colonization.

## DISCUSSION

The aim of this study was to investigate the effect of oral care with CHX solution, HEX solution, and NS solution on the oral health status of critically ill, intubated patients. The most favorable results were obtained with CHX, followed by HEX. The application of the CHX solution resulted in a statistically significant reduction in the number of patients colonized by *Streptococcus, Staphylococcus*, and *Klebsiella* from day 4 to day 7 compared to HEX and NS solutions.

We found highly significant differences between the CHX and the NS group, as well as between the HEX and the NS group in the reduction of oropharyngeal and endotracheal Gram-negative microorganisms. The CHX solution had a greater effect on reducing Gram-positive microorganisms than Gram-negative microorganisms. On day 7, there were significant differences in the isolation of *Klebsiella* and *Staphylococcus aureus* between the CHX and the NS group and between the HEX and the NS group, in favor of the CHX and NEX solutions, respectively. Oropharyngeal colonization occurred rarely after tracheal colonization; most frequently, it occurred concurrently or before it, especially in the NS group.

The orally intubated patient is at great risk of colonization by microorganisms; owing to the presence of salivary disturbances, mucosal desiccation, and mechanical injuries, fixation tapes quickly become heavily contaminated with pathogens [16,17]. Research has found CHX, a topical antibiotic mouth rinse that is effective against both Gram-positive and Gram-negative microorganisms, to be effective in preventing VAP. According to these studies, using 0.12% CHX solution significantly reduced the incidence of pneumonia, nosocomial infection rates, and Gram-negative microorganisms. CHX also has a strong antibacterial effect and decreases the rates of nosocomial pathogen resistance [18,19].

Considering the potential benefits in mitigating gingival and plaque inflammation, HEX does not appear to be an adequate substitute for CHX [20]. With its 80% reduction in plaque-causing microorganisms and its bactericidal and bacteriostatic properties against oral bacteria, CHX works fast, has low toxicity, and is efficient against oral germs [21]. Being a positively charged cationic substance, CHX adheres strongly to the bacterial cell wall and alters the structural integrity of the bacterial cell membrane. When free CHX molecules enter the cells, they induce protein coagulation, thereby reducing cellular activity, which ultimately leads to cell death [22]. When combined with other substances like Moraceae or used alone, CHX significant reduces the level of bacteria in the saliva [23].

Regarding the oral health status of the patients from all three groups, we observed a great degree of similarity between the initial average scores of the oral assessment and those obtained on day 4. This supports the idea that antiseptic solutions do not have any effect in the first 7 days. This finding is in line with the study of Hassan *et al*. [24], which found that antiseptic oral rinses should be used for 7–16 days to provide their optimum antibacterial effect. In addition, after the oral assessment performed on day 7, the study groups had significantly lower scores than the control group. A lower score or a mild alteration of the lips, tongue, saliva, mucous membrane, gingiva, and teeth can indicate that the CHX and HEX solutions improved oral hygiene. This observation is in accordance with the study conducted by Ćabov *et al*. [25], who stated that oral decontamination with CHX significantly decreased oropharyngeal colonization. Handa *et al*. [26] revealed that an oral care protocol that included mouthwashes was effective in improving oral health assessment scores and the oral microbiological flora of hospitalized children by reducing oral infections and bacterial colonization.

We also observed a significant improvement in the oral health status of patients in the CHX group on day 7 of observation. These findings are supported by Silvestri *et al*. [27], who found that the use of CHX in critically ill patients improves their oral health and significantly reduces the incidence of nosocomial pneumonia and VAP. A systematic review of 17 studies [28] showed that the use of CHX for oropharyngeal decontamination lowers the incidence of VAP.

Regarding oropharyngeal colonization with different bacterial species, we observed that on admission, the oropharynx was colonized in all patients of the study and control groups. This finding is in line with those of Daneman *et al*. [29], who reported that on admission to the ICU, the oropharynx was colonized in 81% of the study groups and 70% of the control group. In our study, the most frequently isolated species in the study and control groups were *Streptococcus* and *Staphylococcus aureus*. Similar results were reported by Cojocaru *et al*. [30], who found that the most frequently isolated microorganisms from the oropharynx during the first day of mechanical ventilation were Gram-positive cocci.

Similarly to the results reported by Sole *et al*. [31], cultures taken three times per week from orally intubated patients revealed that colonization of the oropharynx progressed rapidly. However, in the study groups, there was a reduction in the number of patients with bacterial colonization from day 4 to day 7. This reduction may be caused by the antiseptic effect of CHX and HEX against both Gram-positive and Gram-negative bacteria. Another study found that when used daily in addition to regular oral hygiene practices, mouthwashes containing CHX or HEX can lower oral bacterial load counts in healthy individuals, and that CHX has a stronger inhibitory effect on oral germs than HX. However, this study was conducted on healthy subjects rather than patients admitted to the ICU [32].

As far as tracheal colonization is concerned, we found a significant reduction in the number of patients colonized with tracheal Gram-positive organisms in the CHX group, whereas tracheal Gram-negative organisms were more frequently isolated in the control group than in the study groups. There was a significant difference between the CHX and the NS group, as well as between the HEX group and the NS group regarding the isolation of endotracheal Gram-negative microorganisms. This could be attributed to the stronger antiseptic effect of CHX and HEX compared to NS [12].

We also found that oropharyngeal colonization preceded tracheal colonization in the majority of the control group and it occurred at the same time of tracheal colonization in more than half of the control group and considerable percentages in the study groups. This indicates that poor dental hygiene can be linked to respiratory pathogen colonization. In a study conducted by Garrouste-Orgeas *et al*. [33], most patients had bacterial colonization of the oropharynx, and microorganisms isolated from the oropharynx before a diagnosis of pneumonia were identical to the pathogen that caused pneumonia. This finding is consistent with our results and validates the hypothesis that decreasing tracheal and oropharyngeal colonization would contribute to a decrease in the incidence of pneumonia.

Furthermore, we found a concordance between microorganisms infecting the trachea and oropharyngeal flora in the study and control groups. This could be explained as the endotracheal tube bypassing natural defenses, destroying tracheal cilia, and allowing oral pharyngeal fluids to enter the trachea and lower airways. As reported by Sole *et al*. [31], the pathogen responsible for 66% of 35 episodes of respiratory infection was the same as the one that had initially colonized the oral cavity.

A limitation of our study is that it was limited to critically ill patients with endotracheal tubes attached to mechanical ventilators or an oxygen source. Therefore, further studies are needed to explore to what extent the reduction of oropharyngeal and tracheal colonization would reduce the occurrence of VAP.

## CONCLUSION

The findings of this study highlight the importance of performing oral hygiene for critically ill patients, especially for those with endotracheal tubes. The study revealed that CHX has a more powerful effect than HEX and NS in improving the oral mucosa and decreasing the colonization of both the oropharynx and the trachea. Furthermore, compared to other solutions, mouthwashes containing CHX have a higher inhibitory effect on oral germs. Consequently, using CHX mouthwash at standard concentrations is a sufficient and secure method for maintaining oral hygiene that can reduce the risk of VAP in critically ill patients.

## Data Availability

Further data are available from the corresponding author upon reasonable request.
